# A Potent Single-Domain Antibody Targeting LAG-3 for Efficient Tumor Immunotherapy

**DOI:** 10.3390/cimb48050478

**Published:** 2026-05-04

**Authors:** Mengfei Dong, Wenjie Li, Tailin Wang, Ming Li, Jingyi Zhang, Xianglei Liu

**Affiliations:** 1 National Key Laboratory of Lead Druggability Research, Shanghai Institute of Pharmaceutical Industry Co., Ltd., China State Institute of Pharmaceutical Industry, Shanghai 201203, China; 2 School of Pharmaceutical Sciences, Shanghai Jiao Tong University, Shanghai 200240, China; 3 Department of Basic Medical Sciences, Changsha Health Vocational College, Changsha 410600, China

**Keywords:** LAG-3 (CD223), single-domain antibody (sdAb), fibrinogen-like protein 1 (FGL1), immune checkpoint, tumor immunotherapy

## Abstract

Lymphocyte activation gene-3 (LAG-3) is a pivotal immune checkpoint receptor that exerts a negative regulatory effect on T-cell function. Although LAG-3-blocking antibodies have shown promising clinical potential, the inherent limitations of conventional monoclonal antibodies necessitate the development of novel antibody formats with enhanced biological and pharmacological properties. In this study, a panel of single-domain antibodies (sdAbs) targeting human LAG-3 was generated via phage display technology. Among these candidates, 2H-G7 was identified as a high-affinity sdAb that binds to LAG-3 with an equilibrium dissociation constant (K_D_) in the nanomolar range. Notably, 2H-G7 potently blocks the interactions of LAG-3 with both of its key ligands, fibrinogen-like protein 1 (FGL1) and major histocompatibility complex class II (MHC-II). Its capacity to restore impaired T-cell function was validated by quantifying interleukin-2 (IL-2) secretion and CD69 expression in stimulated primary human peripheral blood mononuclear cells (PBMCs). Epitope mapping studies localized the binding site of 2H-G7 to the D1D2 extracellular domains of LAG-3, distinct from relatlimab, a clinically approved LAG-3-blocking antibody serving as the benchmark. In a xenogeneic mouse model of non-small-cell lung cancer (NSCLC), 2H-G7-Fc exhibited superior tumor growth inhibition efficacy compared with relatlimab. These findings demonstrate that 2H-G7 is a promising lead candidate for the development of next-generation LAG-3-targeted tumor immunotherapies.

## 1. Introduction

Immune checkpoint blockade has revolutionized the landscape of cancer therapy; however, a substantial proportion of patients either fail to respond to treatment or develop acquired resistance. This clinical dilemma underscores the urgent need to target alternative inhibitory immune pathways to improve therapeutic outcomes [1]. Lymphocyte Activation Gene-3 (LAG-3, CD223) has emerged as a pivotal co-inhibitory receptor in this context. Originally identified on activated human T cells and natural killer (NK) cells, LAG-3 is now recognized as a key mediator of T-cell exhaustion and is widely expressed on various immune cells within the tumor microenvironment (TME) [2,3]. Structurally, LAG-3 belongs to the immunoglobulin superfamily (IgSF) and shares significant sequence homology with CD4. A distinctive structural feature is an additional loop within its membrane-distal D1 domain, which advanced structural analyses have recently identified as indispensable for high-affinity ligand binding and subsequent immune regulation [4,5]. The clinical relevance of LAG-3 is highlighted by its frequent upregulation on tumor-infiltrating lymphocytes (TILs) in multiple malignancies, including non-small-cell lung cancer (NSCLC). In such cancers, LAG-3 expression is closely associated with the suppressed effector function of TILs and poor clinical prognosis in patients [6,7].

The immunosuppressive function of LAG-3 is mediated through its interactions with multiple ligands. The classical ligand, MHC-II, binds to LAG-3 with a significantly higher affinity than it does to CD4. Recent mechanistic studies have elucidated that this interaction is highly conformation-dependent, enabling LAG-3 to competitively inhibit CD4-mediated T-cell co-stimulation and directly transduce inhibitory intracellular signals, thereby dampening T-cell activation and proliferation [8,9]. A transformative discovery in LAG-3 biology identified fibrinogen-like protein 1 (FGL1), a hepatocyte-secreted soluble factor, as a major high-affinity ligand of LAG-3 [10]. The FGL1-binding site on LAG-3 has been precisely mapped through mutagenesis studies that identified amino acid mutations altering hLAG-3 affinity for FGL1 [11]. The FGL1-LAG-3 signaling axis functions independently of the MHC-II-LAG-3 pathway and has been functionally validated as a key mechanism underlying tumor immune escape in multiple preclinical cancer models [12,13]. The existence of these two distinct ligand-binding pathways highlights the complex biological regulation of LAG-3 and presents unique therapeutic opportunities for targeted intervention.

Clinically, targeting LAG-3 has proven to be a viable and effective therapeutic strategy, as exemplified by the US Food and Drug Administration (FDA) approval of relatlimab—a human monoclonal antibody that inhibits LAG-3 binding to both MHC-II and FGL1—in combination with nivolumab for the treatment of metastatic melanoma [14]. Despite this clinical success, conventional monoclonal antibodies (mAbs) possess inherent limitations, including a large molecular weight (~150 kDa) that hinders penetration into solid tumor tissues, complex and costly manufacturing processes, and potential immunogenicity in patients. These drawbacks have driven extensive research into next-generation antibody formats with improved properties [15,16]. Single-domain antibodies (sdAbs, also known as nanobodies) represent a promising alternative platform for developing robust immune checkpoint inhibitors [17,18], owing to their unique advantages such as a small molecular weight (~15 kDa), superior tissue and tumor penetration, high thermal and structural stability, and ease of genetic engineering and manufacturing. Developing high-affinity sdAbs that specifically block LAG-3-ligand interactions may therefore yield potent therapeutic agents with favorable pharmacological properties for cancer immunotherapy.

In this study, we panned a naïve phage display library against the human LAG-3 protein to isolate specific sdAbs. Positive clones were subsequently evaluated using competitive binding assays to assess their ability to block LAG-3 interactions with FGL1 and MHC-II, and their capacity to restore T-cell function was measured via IL-2 secretion and CD69 expression assays [19]. Finally, the in vivo anti-tumor efficacy of the lead candidate 2H-G7 was evaluated in a PBMC-humanized mouse model of NSCLC. Our results demonstrate that 2H-G7 is a novel and potent LAG-3 antagonist with great potential for the development of next-generation LAG-3-targeted cancer immunotherapies.

## 2. Materials and Methods

### 2.1. Cell Lines

Cell lines used for functional assays were obtained and cultured as follows: Raji (human Burkitt’s lymphoma, Cat: TCHu 44) cell lines were purchased from the Cell Bank of the Chinese Academy of Sciences (Shanghai, China) and cultured in RPMI-1640 medium (Gibco, Thermo Fisher Scientific, Waltham, MA, USA, Cat: 11875093) supplemented with 10% fetal bovine serum (FBS, NCM biotech, Shanghai, China, Cat: C9500). The A549 (Jinyuan Biotechnology, Shanghai, China, Cat: JY135) human non-small-cell lung cancer cell line was maintained in RPMI-1640 medium containing 10% FBS. Expi293F human cells (Thermo Fisher Scientific, Waltham, MA, USA, Cat: A14527CN) were cultured in OPM-293 CD05 Medium (OPM Biosciences, Shanghai, China, Cat: 81075-001). The HEK-293T (Jinyuan Biotechnology, Shanghai, China, Cat: JY759) cell line was cultured in DMEM (Shanghai Zhong Qiao Xin Zhou Biotechnology, Shanghai, China, Cat: ZQ-100) containing 10% FBS. The HEK^-LAG3-EGFP^ cell line was generated in-house by stably transfecting parental HEK-293T cells with an LAG3-EGFP expression construct. Primary human peripheral blood mononuclear cells (PBMCs) were obtained from Shanghai Miaoshun Biotech Co., Ltd, Shanghai, China. The human PBMC samples used in this study were approved by the Ethics Committee of Shanghai Liquan Hospital Institutional Review Board (Approval No.: 2021-01), and all donors provided written informed consent. The CHO-K1 (Chinese hamster ovary, Jinyuan Biotechnology, Shanghai, China, Cat: JY210) cell line was cultured in F-12K medium (Gibco, Thermo Fisher Scientific, Waltham, MA, USA, Cat: 21127022) supplemented with 10% FBS (NCM biotech, Shanghai, China, Cat: C9500). All cell lines were incubated at 37 °C in a humidified atmosphere containing 5% CO_2_ for all functional assays.

### 2.2. Phage Panning

Recombinant human LAG-3-mFc protein (Sino Biological, Beijing, China, Cat: 16498-H05H) was immobilized on microtiter plates for phage library selection. A naïve sdAb phage display library was incubated with the immobilized LAG-3-mFc antigen, followed by extensive washing with PBST (PBS containing 0.05% Tween-20) to remove non-specifically bound phages. Specifically bound phages were eluted with 0.2 M glycine-HCl (pH 2.2), and the eluate was immediately neutralized with 1 M Tris-HCl (pH 8.0). The neutralized eluate was used to infect TG1 (Lucigen, LGC Biosearch Technologies, Novato, CA, USA, Cat: 605021) competent *Escherichia coli* cells for phage propagation and amplification. A total of three rounds of phage panning were performed, with the washing stringency gradually increased in subsequent rounds to enrich for high-affinity binders. Enriched phages from the final panning round were amplified in 2× YT medium containing ampicillin (Beyotime, Shanghai, China, Cat:ST005) and helper phages (NEB, Ipswich, MA, USA, Cat: N0315S). Individual phage clones were then screened for specific LAG-3 binding by enzyme-linked immunosorbent assay (ELISA). Plates were coated with LAG-3-mFc or bovine serum albumin (BSA, Biosharp, Beijing, China, Cat: BS114, as a negative control), blocked to eliminate non-specific binding, and incubated with phage supernatants from individual clones. Specific phage binding was detected using an anti-M13 monoclonal antibody (Sino Biological, Beijing, China, Cat: 11973-MM05T-H) and 3,3′,5,5′-tetramethylbenzidine (TMB, NCM biotech, Shanghai, China, Cat: M30100) chromogenic substrate. Positive clones with specific LAG-3-binding activity were sequenced to obtain their sdAb nucleotide sequences for further protein expression and functional characterization.

### 2.3. ELISA for Antibody Binding Assay

To quantitatively assess the binding of selected sdAbs to LAG-3, an indirect ELISA was performed. ELISA plates were coated with 100 μL/well of recombinant human LAG3-Fc protein (Sino Biological, Beijing, China, Cat: 16498-H02H) diluted to a concentration of 1 μg/mL in PBS and incubated at 4 °C overnight. After three washes with PBST, non-specific binding sites were blocked with 3% Blotting-Grade Blocker (Bio-Rad, Hercules, CA, USA, Cat: 1706404) in PBS at 37 °C for 60 min. Purified sdAbs were serially diluted 3-fold from an initial concentration of 1000 nM across eight gradients, added to the coated plates, and incubated at 37 °C for 60 min. Following five washes with PBST, HRP-conjugated anti-FLAG secondary antibody (GenScript, Nanjing, China, Cat: A01428) was added to each well and incubated for 1 h at 37 °C. After a final round of five washes with PBST, TMB substrate was added for color development at 37 °C for 15 min, and the chromogenic reaction was terminated by adding 2 M HCl. The absorbance of each well was measured at 450 nm using a microplate reader (Agilent, BIOTEK SYNERGY, Santa Clara, CA, USA). An irrelevant non-LAG3-binding antibody (Sino Biological, Beijing, China, Cat: 10004-MM01) was used as the negative control in all assays.

### 2.4. Flow Cytometry Assay

To verify the binding of sdAbs to cell surface-expressed LAG-3, a flow cytometry assay was performed. HEK293 cells were transiently transfected with a pcDNA3.1-based expression vector containing the full-length human LAG3 gene to generate HEK^-LAG3-EGFP^ cells, and transfection efficiency was confirmed by EGFP fluorescence at 48 h post-transfection. Transfected HEK^-LAG3-EGFP^ cells were harvested, and cell suspensions (5 × 10^5^ cells per sample in 100 μL PBS) were incubated with test sdAbs at a final concentration of 1 μM on ice for 2 h. Cells were then washed twice with cold PBS and incubated with Alexa-488-conjugated anti-FLAG secondary antibody (GenScript, Nanjing, China, Cat: A01809) on ice for 1 h in the dark to avoid fluorescence quenching. After two additional washes with cold PBS, cells were resuspended in 100 μL PBS, and the mean fluorescence intensity (MFI) was measured using a flow cytometer (Cytek, Northern Lights, Wuxi, China). Untransfected HEK293 cells served as the blank control, an irrelevant non-LAG3 antibody (Sino Biological, Beijing, China, Cat: 10004-MM01) as the negative control, and relatlimab-scFv expressed by our lab as the positive control.

### 2.5. Biolayer Interferometry (BLI)

The binding kinetics and affinity of selected sdAbs to LAG-3 were quantitatively analyzed using a BLI system (Gator Plus, Gator Bio, Suzhou, China). Recombinant human LAG3-mFc protein (Sino Biological, Beijing, China, Cat: 16498-H05H) was immobilized onto anti-human Fc capture biosensor tips (Gator Bio, Suzhou, China, Cat: 20-5036) for 120 s to form a stable antigen coating. Purified sdAbs were serially diluted 3-fold from an initial concentration of 1000 nM across five concentrations and used as analytes to assess binding to the immobilized LAG3-mFc. The association phase and dissociation phase of the antigen–antibody interaction were each monitored for 300 s. All BLI experiments were performed at 25 °C with constant shaking. The obtained binding sensorgrams were fitted to a 1:1 Langmuir binding model using the Gator Bio analysis software (version 2.18.7.0718) to calculate the equilibrium dissociation constant (K_D_), association rate constant (k_a_), and dissociation rate constant (k_d_).

### 2.6. Competitive ELISA for LAG3-FGL1 Blockade Assay

A competitive ELISA was established to assess the ability of sdAbs to block the interaction between LAG-3 and FGL1. 96-well ELISA plates were coated overnight at 4 °C with 100 μL/well of recombinant human FGL1 protein (Sino Biological, Beijing, China, Cat: 13484-H08B) diluted to 1 μg/mL in PBS. After coating, plates were blocked with 5% BSA in PBS at 37 °C for 60 min and washed three times with PBST. Test sdAbs (at a final concentration of 1000 nM) were pre-mixed with recombinant LAG3-mFc protein (at a final concentration of 100 nM), and the mixture was added to the FGL1-coated plates and incubated at 37 °C for 60 min. Plates were then washed five times with PBST to remove unbound proteins, and HRP-conjugated AffiniPure Goat Anti-Mouse IgG (H + L) (Jackson Immuno Research, West Grove, PA, USA, Cat: 115-035-003) was added and incubated at 37 °C for 45 min. After five additional washes with PBST, TMB substrate was added for color development at 37 °C for 15 min, the reaction was stopped with 2 M HCl, and absorbance was measured at 450 nm. The inhibition of LAG3-FGL1 binding by sdAbs was calculated by comparing the absorbance of test samples with that of the negative control group.

### 2.7. Competitive Flow Cytometry for LAG3-MHC-II Blockade Assay

A competitive flow cytometry assay was used to evaluate the ability of sdAbs to block the interaction between LAG-3 and MHC-II. Raji cells, which endogenously express high levels of MHC-II (Cell Bank of the Chinese Academy of Sciences, Shanghai, China, Cat: TCHu 44), were collected by centrifugation and resuspended at a density of 3 × 10^5^ cells per sample in 100 μL cold PBS. Test sdAbs were serially diluted 3-fold from an initial concentration of 1 μM and pre-incubated with the Raji cell suspension on ice for 1 h. After two washes with cold PBS to remove unbound antibodies, recombinant human LAG3-mFc protein (diluted to 200 nM) was added to the antibody-pre-incubated cells and further incubated on ice for 1 h. Cells were then washed twice with cold PBS and stained with Alexa-647-conjugated anti-mouse Fc secondary antibody (BioLegend, San Diego, CA, USA, Cat: 405322) on ice for 1 h in the dark. Following two final washes with cold PBS, cells were resuspended in 200 μL PBS, and the MFI was analyzed by flow cytometry. An irrelevant non-LAG3 antibody (Sino Biological, Beijing, China, Cat: 10004-MM01) was used as the negative control, and the inhibition of LAG3-MHC-II binding was determined by comparing the MFI of test groups with that of the negative control group.

### 2.8. T Cell Activation Assay

The biological activity of anti-LAG3 sdAbs was evaluated by measuring their ability to restore T-cell activation, as assessed by IL-2 secretion and CD69 expression in staphylococcal enterotoxin B (SEB, Toxin Technology, Sarasota, FL, USA, Cat: BT202)-stimulated primary human PBMCs. Human PBMCs were isolated from fresh peripheral blood samples using density gradient centrifugation and resuspended in complete RPMI-1640 medium containing SEB (a T-cell mitogen). The PBMC suspension was seeded into 24-well plates, and murine anti-LAG3 sdAbs or the positive control antibody relatlimab-scFv were diluted to a target concentration of 1 μM in complete RPMI1640 medium and added to the corresponding wells. Plates were then incubated at 37 °C in a 5% CO_2_ atmosphere for 5 days. After incubation, cell culture supernatants were collected by centrifugation, and the concentration of IL-2 in the supernatants was quantified using a commercial ELISA kit (Beyotime, Shanghai, China, Cat: PI580) according to the manufacturer’s instructions. Additionally, PBMCs were harvested from the wells, washed with cold PBS, and stained with FITC anti-human CD69 Antibody (BioLegend, San Diego, CA, USA, Cat: 310904) for flow cytometry analysis to determine the expression of CD69.

### 2.9. Epitope Mapping

To identify the binding epitope of the lead sdAb 2H-G7 on LAG-3, truncated forms of LAG-3 containing extracellular domain D1D2 were constructed, heterologously expressed, and purified. The purity and molecular weight of the truncated LAG-3 proteins were verified by SDS-PAGE. An indirect ELISA was then performed to assess the binding of 2H-G7 and a positive control anti-LAG3 antibody to the truncated protein LAG3-D1D2. Plates were coated with the various LAG-3 proteins, blocked, and incubated with 2H-G7 or the positive control antibody. Specific binding was detected with HRP-conjugated anti-human IgG (AlpVHHs, Chengdu, China, Cat: 023-112-005) or anti-DYKDDDDK tag (AlpVHHs, Chengdu, China,, Cat: 016-303-005) secondary antibody and TMB substrate, and absorbance was measured at 450 nm. The binding epitope of 2H-G7 was inferred based on the binding activity of the sdAb to the different LAG-3 truncation mutants.

### 2.10. Competitive ELISA for 2H-G7 to Relatlimab Binding to LAG-3

A competitive ELISA was established to assess the ability of 2H-G7 to block the interaction between LAG-3 and relatlimab. 96-well ELISA plates were coated overnight at 4 °C with 100 ng/well of recombinant human LAG-3-His protein (Sino Biological, Beijing, China, Cat: 16498-H08H). After coating, plates were blocked with 5% BSA in PBS at 37 °C for 60 min and washed three times with PBST. 2H-G7 (Flag tag) was serially diluted 3-fold from an initial concentration of 500 nM and pre-mixed with 3 nM relatlimab (Fc tag), and the mixture was added to the LAG-3-coated plates and incubated at 37 °C for 60 min. Plates were then washed five times with PBST to remove unbound proteins, and HRP-conjugated anti-human IgG antibody (AlpVHHs, Chengdu, China, Cat: 023-112-005) was added and incubated at 37 °C for 45 min. On the contrary, relatlimab (Fc tag) was serially diluted 3-fold from an initial concentration of 500 nM were pre-mixed with 500 nM 2H-G7 (Flag tag), and the mixture was added to the LAG-3-coated plates and incubated at 37 °C for 60 min. Plates were then washed five times with PBST to remove unbound proteins, and HRP-conjugated anti-Flag antibody (GenScript, Nanjing, China, Cat: A01428) was added and incubated at 37 °C for 45 min. After five additional washes with PBST, TMB substrate was added for color development at 37 °C for 15 min, the reaction was stopped with 2 M HCl, and absorbance was measured at 450 nm.

### 2.11. Protein–Protein Docking Between Human LAG3 and 2HG7

Protein–protein docking between human LAG3 and 2HG7 was performed using the HADDOCK2.4 web server [20]. Based on the existing X-ray structure of LAG3 together with the AlphaFold3-predicted structure, surface-exposed candidate epitope residues within the D1 and D2 regions of LAG3 were designated as active or passive residues. 2HG7 residues located within the VH complementarity-determining regions (CDRs) were defined as active residues, and neighboring surface-accessible residues were assigned as passive residues. After these inputs were provided, HADDOCK2.4 automatically generated interaction restraints and performed docking according to the default workflow. Upon completion of docking, the resulting models were clustered following the standard HADDOCK2.4 procedure, and the clusters were ranked according to HADDOCK score. The candidate conformation with the best HADDOCK score was selected and superimposed with the X-ray structure of relatlimab (PDB:7UM3) based on the shared LAG-3 region for visual comparison of their potential binding sites and spatial relationships.

### 2.12. Antibody Stability Analysis

The stability of antibodies was assessed using Prometheus Panta (NANOTEMPER, Munich, Germany). The antibody concentration was adjusted to 1 mg/mL, loaded into capillaries, and measured in triplicate. The thermal unfolding experiment was initiated at 25 °C, increasing at a rate of 2 °C/min until reaching 100 °C. Fluorescence intensities at wavelengths of 330 nm and 350 nm were simultaneously recorded throughout the heating process. Additionally, back-reflection measurements were performed to monitor sample turbidity changes. All data were collected and subsequently analyzed using the PR.Panta analysis software (version 1.8) to determine the melting temperature (T_m_) and the onset temperature of turbidity (T_turbidity_).

### 2.13. Animal Experiments

A humanized immunodeficient mouse model was established to evaluate the in vivo anti-tumor efficacy of 2H-G7. Four- to six-week-old female huPBMC-NOG-dKO immunodeficient mice (15, Beijing Vital River, Beijing, China) were subcutaneously inoculated in the right posterior flank with 5 × 10^6^ A549 NSCLC cells suspended in 100 μL of serum-free medium. One week after tumor cell inoculation, 5 × 10^6^ human PBMCs were administered to each mouse via tail vein injection to reconstitute a humanized immune system. Successful engraftment of human PBMCs was confirmed by flow cytometric analysis of peripheral blood collected two weeks post-PBMC injection. When subcutaneous tumor volumes reached 100–150 mm^3^, mice were randomly assigned into three experimental groups (*n* = 5 mice per group) to ensure uniform initial tumor sizes across groups. Mice were then treated via tail vein injection with 2H-G7-Fc, the reference antibody relatlimab (Sino biological, Beijing, China, Cat: 68142-H001), or PBS (vehicle control) every four days for a total duration of 8 weeks. Tumor dimensions (length and width) were measured weekly using a digital caliper, and tumor volume was calculated using the formula: V = (length × width^2^)/2. Body weight and survival status of the mice were monitored weekly to assess treatment-related toxicity and perform survival analysis, respectively. Mice were euthanized at the experimental endpoint, and subcutaneous tumors were excised, weighed, and processed for subsequent analyses including snap-freezing in liquid nitrogen, histopathological examination, and cryopreservation for further molecular studies. Animal experimental protocols were approved by the Institute Ethics Committee of the Shanghai Institute of Pharmaceutical Industry (A-2024-10-01), and all procedures strictly followed the approved protocols.

### 2.14. Analysis of Tumor-Infiltrating Effector T Cells

At the study endpoint, tumors were excised, mechanically dissociated into 1–3 mm^3^ fragments, and enzymatically digested to prepare single-cell suspensions. Cell suspensions were passed through a 70 μm nylon mesh filter before staining. Cells were incubated with anti-CD45-APC (Sino biological, Beijing, China, Cat: 10086-MM05-A), anti-CD4-488 (Thermo Fisher, Waltham, MA, USA, Cat: 53-0049-42), and anti-CD8-PE (Sino biological, Beijing, China, Cat: 10980-MM48-P) antibodies for 30 min on ice in the dark, followed by three washes with PBS supplemented with 2% FBS. Flow cytometric data were acquired on a flow cytometer, and CD45+ leukocytes were gated before analysis of CD4+ and CD8+ T-cell populations. Data were analyzed using FlowJo v10.

### 2.15. Pharmacokinetic (PK) Analysis

To evaluate the in vivo pharmacokinetic (PK) profiles of the antibodies, Balb/C mice (*n* = 3 per group) received a single intravenous injection of 2H-G7-Fc or relatlimab (Sino biological, Beijing, China, Cat: 68142-H001) at 10 mg/kg. Peripheral blood samples were collected at 0.5, 1, 2, 4, 6, 8, 12, 48, 72, and 96 h after administration, and serum was isolated for subsequent antibody quantification. Serum antibody concentrations were determined by ELISA. Briefly, individual wells of a 96-well half-area immunoplate were coated with 100 ng/well recombinant human LAG-3 antigen. After blocking with 3% BSA in PBS, serially diluted standards and serum samples were added and incubated for binding. Bound antibodies were detected using an HRP-conjugated anti-human Fc (AlpVHHs, Chengdu, China, Cat: 023-112-005) secondary antibody, and serum concentrations at each time point were calculated based on the corresponding standard curve. Pharmacokinetic parameters, including serum half-life (t_1/2_), were subsequently calculated using a noncompartmental analysis (NCA) model.

### 2.16. Statistical Analysis

All experimental data are presented as the mean ± standard deviation (SD) unless otherwise specified. Normality of data distribution was assessed using the Shapiro–Wilk test prior to statistical testing. For comparisons between two groups, an unpaired Student’s *t*-test was used if data were normally distributed; otherwise, a nonparametric Mann–Whitney U test was applied. For multiple group comparisons, one-way analysis of variance (ANOVA) followed by post hoc tests was used for normally distributed data, while the Kruskal–Wallis test was used for non-normally distributed data. A *p* value < 0.05 was considered statistically significant for all analyses. All statistical tests were performed using GraphPad Prism software (Version 9.0).

## 3. Results

### 3.1. Characterization of Anti-LAG3 Single-Domain Antibodies

**Figure 1:**
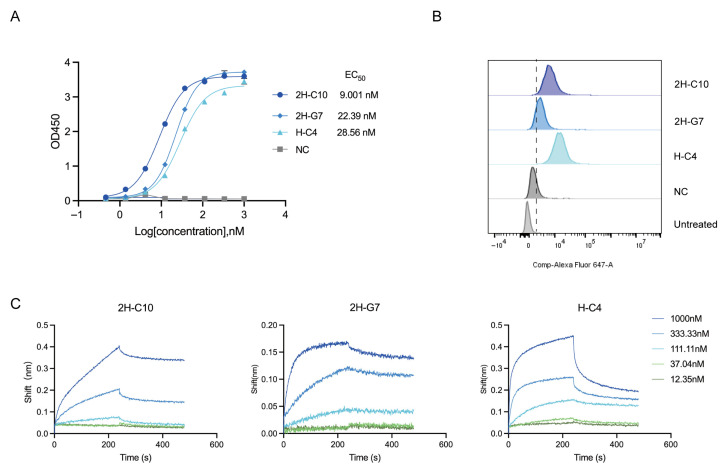
Evaluation of the binding characteristics of anti-LAG3 single-domain antibodies. (**A**) Dose-dependent binding of the three sdAbs (2H-C10, 2H-G7, H-C4) to recombinant human LAG-3 protein as assessed by indirect ELISA (*n* = 3). (**B**) Specific binding of the sdAbs to cell surface LAG-3 on HEK^-LAG3-EGFP^ cells as detected by flow cytometry; MFI values reflect the binding intensity. (**C**) Binding kinetics of the sdAbs to LAG-3 measured by BLI; sensorgrams show dose-dependent binding, and K_D_ values were calculated from the 1:1 binding model fitting.

To isolate human LAG-3-specific single-domain antibodies (sdAbs), we performed four rounds of phage panning against recombinant human LAG-3 protein using a fully human sdAb phage display library, which yielded more than 100 individual phage clones with potential LAG-3-binding activity. Initial screening by indirect ELISA identified three positive clones, 2H-C10, 2H-G7, and H-C4, all of which exhibited specific and dose-dependent binding to LAG-3, with EC_50_ values of 9.001 nM, 22.39 nM, and 28.56 nM, respectively (Figure 1A). As the first approved fully human IgG4 antibody targeting LAG-3, relatlimab was simultaneously expressed and included as a positive control. ELISA analysis showed that relatlimab bound LAG-3 with an EC_50_ of 3.11 nM (Figure S1A). Flow cytometry analysis further confirmed that all three sdAbs effectively recognized HEK^-LAG3-EGFP^ cells expressing cell surface LAG-3, as evidenced by significantly increased mean fluorescence intensity (MFI) compared with the negative control antibody (Figure 1B), thereby confirming their specific binding to membrane-expressed LAG-3.

To quantitatively characterize the binding affinities of these candidate sdAbs, we next performed kinetic analysis using biolayer interferometry (BLI). Recombinant human LAG-3-Fc protein was immobilized onto AHC biosensor tips, and the binding kinetics of the three sdAbs were evaluated using serial dilutions. All three sdAbs exhibited clear dose-dependent binding to LAG-3, and fitting of the sensorgrams to a 1:1 binding model yielded the following equilibrium dissociation constants (K_D_) (Figure 1C, Table 1): 7.58 × 10^−8^ M for 2H-C10, 1.99 × 10^−8^ M for 2H-G7, and 3.41 × 10^−8^ M for H-C4. Among these candidates, 2H-G7 displayed the highest binding affinity, with a K_D_ in the low nanomolar range.

Taken together, we successfully identified three human sdAbs with favorable binding affinity and high specificity toward LAG-3 through phage display screening. Among them, 2H-G7 exhibited the strongest binding affinity, supporting its further development as a promising LAG-3-targeting antibody candidate.

### 3.2. Anti-LAG3 sdAbs Effectively Block the Binding of LAG-3 to Its Ligands FGL1 and MHC-II

**Figure 2:**
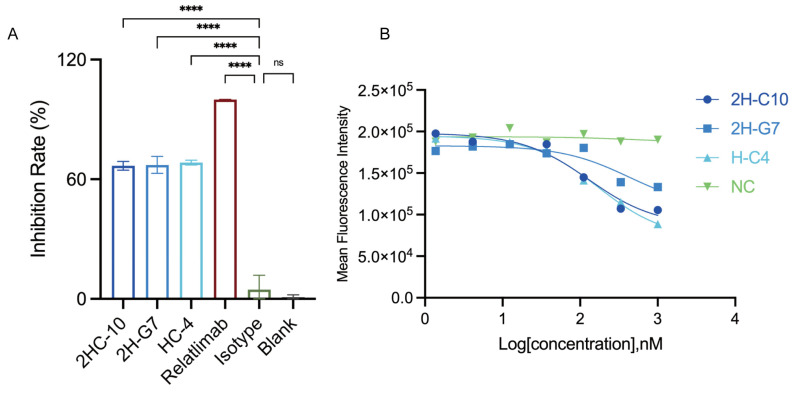
Anti-LAG3 single-domain antibodies block the binding of LAG-3 to its endogenous ligands FGL1 and MHC-II. (**A**) Competitive ELISA to assess the inhibition of human LAG3-FGL1 binding by the three sdAbs; Inhibition rate represents the mean ± SD of triplicate samples, and lower absorbance indicates stronger blocking activity. **** *p* < 0.0001, ns, no significance, Dunnett’s multiple comparisons test. (**B**) Competitive flow cytometry assay to evaluate the inhibition of LAG3-MHC-II binding on Raji cells by the sdAbs; the sdAbs exhibit dose-dependent blockade of the LAG3-MHC-II interaction.

We first assessed the ability of the three candidate sdAbs to block the LAG3-FGL1 interaction using a competitive ELISA. Recombinant human FGL1 protein was coated on ELISA plates, and pre-mixed LAG3-mFc and sdAbs were added to the plates. The amount of LAG3-mFc bound to immobilized FGL1 was detected by HRP-conjugated anti-mouse Fc antibody, and the inhibition efficacy was determined by comparing absorbance with the negative control group. As shown in Figure 2A, all three sdAbs (2H-C10, 2H-G7, H-C4) and relatlimab can effectively inhibit the binding of LAG-3 to immobilized FGL1, with significant reductions in absorbance compared with the negative control, demonstrating their ability to specifically block the LAG3-FGL1 interaction.

We then evaluated the functional blocking activity of the sdAbs against the LAG3-MHC-II interaction using a competitive flow cytometry assay with Raji cells, which endogenously express MHC-II. Raji cells were pre-incubated with serially diluted sdAbs, followed by incubation with LAG3-mFc protein, and the amount of cell-surface-bound LAG3-mFc was detected by Alexa-647-conjugated anti-mouse Fc antibody. As shown in Figure 2B, 2H-C10, 2H-G7, and H-C4 all potently inhibited the binding of LAG-3 to MHC-II on Raji cells in a dose-dependent manner, with significant reductions in MFI compared with the irrelevant negative control antibody (NC), although their inhibitory activity was weaker than that of relatlimab (Figure S2), which binds an epitope that directly overlaps with the MHC-II interface. Collectively, these data identify 2H-C10, 2H-G7, and H-C4 as functional anti-LAG3 sdAbs capable of disrupting both major LAG-3-ligand interactions (LAG3-FGL1 and LAG3-MHC-II).

### 3.3. Anti-LAG3 sdAb 2H-G7 Restores T Cell Activation by Promoting Cytokine Secretion and Activation Marker Expression

To assess the functional biological activity of the anti-LAG3 sdAbs, we measured their ability to enhance T cell activation in SEB-stimulated primary human PBMCs, as evaluated by IL-2 secretion (a key pro-inflammatory cytokine for T cell proliferation and activation) and CD69 expression (an early T cell activation marker). Primary human PBMCs from healthy donors were cultured with SEB and the candidate sdAbs or relatlimab-scFv for 5 days, after which cell culture supernatants were collected for IL-2 quantification by ELISA, and PBMCs were harvested for CD69 expression analysis by flow cytometry.

**Figure 3:**
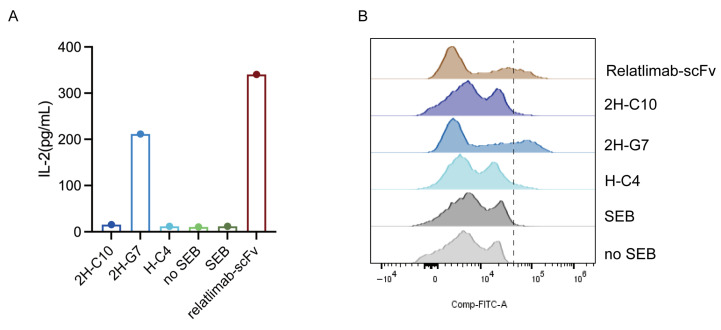
Anti-LAG3 sdAb 2H-G7 restores T cell activation in SEB-stimulated human PBMCs. (**A**) Quantification of IL-2 secretion in PBMC culture supernatants by ELISA (single experiment); 2H-G7 significantly promotes IL-2 secretion compared with the control group. (**B**) Analysis of CD69 expression on PBMCs by flow cytometry; 2H-G7 markedly increases the percentage of CD69-positive activated T cells.

As shown in Figure 3A, in these three sdAbs, only 2H-G7 significantly enhanced IL-2 secretion in SEB-stimulated PBMCs, with a measured IL-2 concentration of 211.4 pg/mL, which was comparable to the positive control relatlimab-scFv (340.6 pg/mL). Inconsistent with the IL-2 secretion results, flow cytometry analysis revealed that 2H-G7 also significantly increased the percentage of CD69-positive cells in the PBMC population (Figure 3B), similar to relatlimab-scFv. These results demonstrate that the lead sdAb 2H-G7 functionally blocks the LAG-3 inhibitory pathway, thereby reversing LAG-3-mediated T cell suppression and restoring T cell activation in human PBMCs.

### 3.4. Epitope Mapping Localizes the 2H-G7 Binding Site to the D1D2 Extracellular Domains of LAG-3

**Figure 4:**
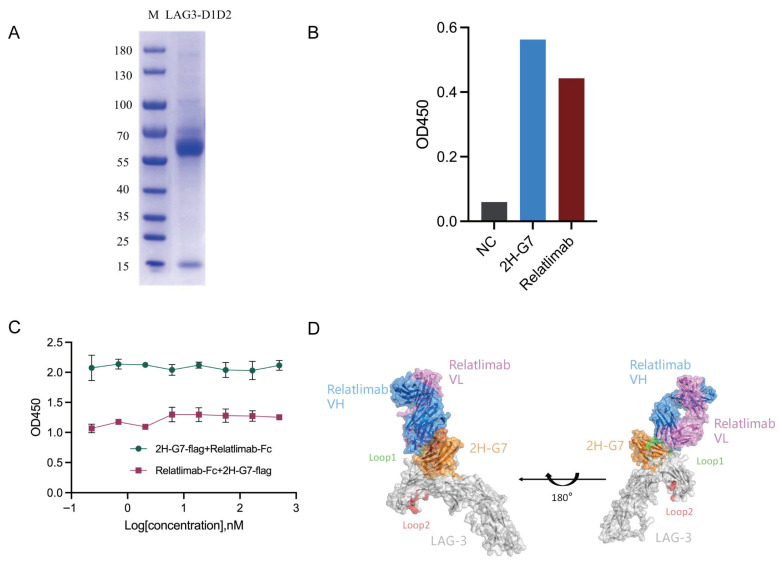
Mapping of the 2H-G7 binding epitope on the extracellular domain D1D2 of LAG-3. (**A**) SDS-PAGE analysis of the purified truncated LAG-3-D1D2 protein. (**B**) Binding of 2H-G7 and a positive control anti-LAG3 antibody to the truncated LAG-3-D1D2 protein by ELISA; 2H-G7 exhibits strong binding to the LAG3-D1D2 domain, indicating its epitope is localized within D1D2. (**C**) Competitive ELISA to assess the inhibition of binding to LAG-3 for relatlimab or 2H-G7; absorbance data represent the mean ± SD of duplicate samples. (**D**) Molecular docking model of 2H-G7 and relatlimab binding to LAG-3. Surface and ribbon representations of the LAG-3 extracellular domain (gray) in complex with 2H-G7 (orange) and relatlimab (blue: VH domain; magenta: VL domain). The two panels show the complex rotated 180° relative to each other, revealing the distinct binding epitopes of the two antibodies. Loop 1 (green) and Loop 2 (red), the primary binding sites for MHC-II and FGL1, respectively, are highlighted. The docking results indicated that the antigen-binding region of 2HG7 was mainly located at residues D46, L58, H63, R143, T150, I164, S170, R171, and P172 within the D1 domain. The core antigen-side recognition region of relatlimab is located within residues H63–W70 of the LAG-3 D1 insertion loop, and overlapping peptide analysis further suggested that its recognition sequence can be mapped to H63PAAPSSW70 [21].

Given its high binding affinity and potent functional activity in restoring T cell activation, 2H-G7 was selected as the lead candidate for further epitope mapping studies to define its binding domain on LAG-3. We constructed and expressed truncated forms of LAG-3 containing extracellular immunoglobulin domains D1D2. The purity and correct expression of this truncated LAG-3 protein was verified by SDS-PAGE (Figure 4A). An indirect ELISA was then performed to assess the binding of 2H-G7 and relatlimab to LAG-3-D1D2 protein. As shown in Figure 4B, 2H-G7 effectively bound to the LAG3-D1D2 truncated protein with a binding intensity comparable to the positive control relatlimab. These results demonstrate that 2H-G7 primarily binds to an epitope located within the D1D2 extracellular domains of LAG-3—the key structural region mediating LAG-3 interactions with FGL1 and MHC-II. To determine the binding epitope relationship between 2H-G7 and relatlimab on LAG-3, competitive binding ELISAs were performed. Serial dilutions of 2H-G7 did not interfere with the binding of relatlimab to immobilized LAG-3 protein. Conversely, graded concentrations of relatlimab also exhibited no inhibitory effect on the binding of 2H-G7 to LAG-3 (Figure 4C). These mutually non-competitive binding patterns clearly indicated that 2H-G7 and relatlimab recognize distinct epitopes within the D1D2 domain of LAG-3. To further validate this observation, molecular docking simulation was conducted. Consistent with the competitive ELISA results, docking analysis revealed that the binding interfaces of 2H-G7 and relatlimab on LAG-3 D1D2 were spatially adjacent but showed only minimal overlap (both involve the H63 amino acid residue, Figure 4D). Relatlimab binds to the Loop 1 (the primary binding sites for MHC-II)-proximal region of LAG-3 D1, while 2H-G7 recognizes a unique, spatially adjacent epitope on the D1D2 interface; notably, the binding site of 2H-G7 is positioned closer to Loop 2 (the primary binding sites for FGL1) compared with relatlimab. Collectively, these results confirmed that 2H-G7 binds to a unique epitope on LAG-3 that is different from that of the clinical antibody relatlimab.

### 3.5. Thermal Stability of 2H-G7 and Relatlimab

**Figure 5:**
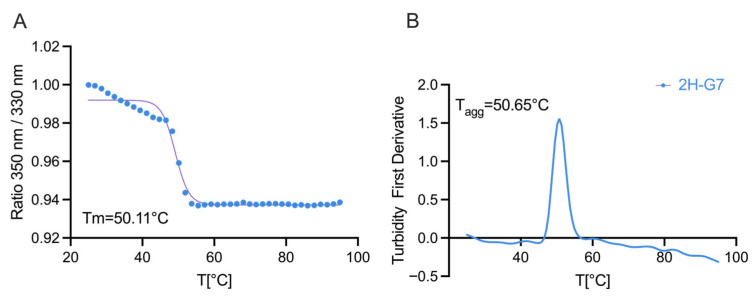
Thermal stability of 2H-G7 as measured by Prometheus Panta (NANOTEMPER). (**A**) Thermal stability of the antibody was assessed by nano differential scanning fluorimetry (nanoDSF) based on intrinsic tryptophan fluorescence. Samples were heated from 20 °C to 100 °C, and the fluorescence intensity ratio (F350/F330) was recorded as a function of temperature. Data represent the mean ± SD of three independent experiments. (**B**) Antibody aggregation was concurrently monitored by backreflection, and the aggregation temperature (Tagg) was determined from the first derivative of the backreflection signal. Data represent the mean ± SD of three independent experiments.

We further evaluated the thermal stability of 2H-G7 in comparison with relatlimab by monitoring changes in intrinsic tryptophan fluorescence during thermal unfolding. The melting temperatures (T_m_) of 2H-G7 and relatlimab were determined to be 50.11 °C and 68.27 °C, respectively (Figure 5A and Figure S1B). In addition, aggregation onset temperatures were observed at 50.65 °C for 2H-G7 and 79.13 °C for relatlimab, indicating that 2H-G7 retained acceptable conformational stability despite its relatively lower thermal robustness compared with the full-length IgG4 antibody relatlimab (Figure 5B and Figure S1B).

### 3.6. In Vivo Anti-Tumor Activity of 2H-G7-Fc in a Humanized NSCLC Mouse Model

To evaluate the in vivo anti-tumor efficacy of the lead sdAb 2H-G7, we constructed a 2H-G7-Fc fusion protein by fusing 2H-G7 to a human IgG1 Fc fragment to prolong its serum half-life and enhance in vivo stability. We then employed a subcutaneous xenogeneic humanized mouse model of non-small-cell lung cancer (NSCLC) using A549 cells and huPBMC-NOG-dKO mice with a reconstituted human immune system.

**Figure 6:**
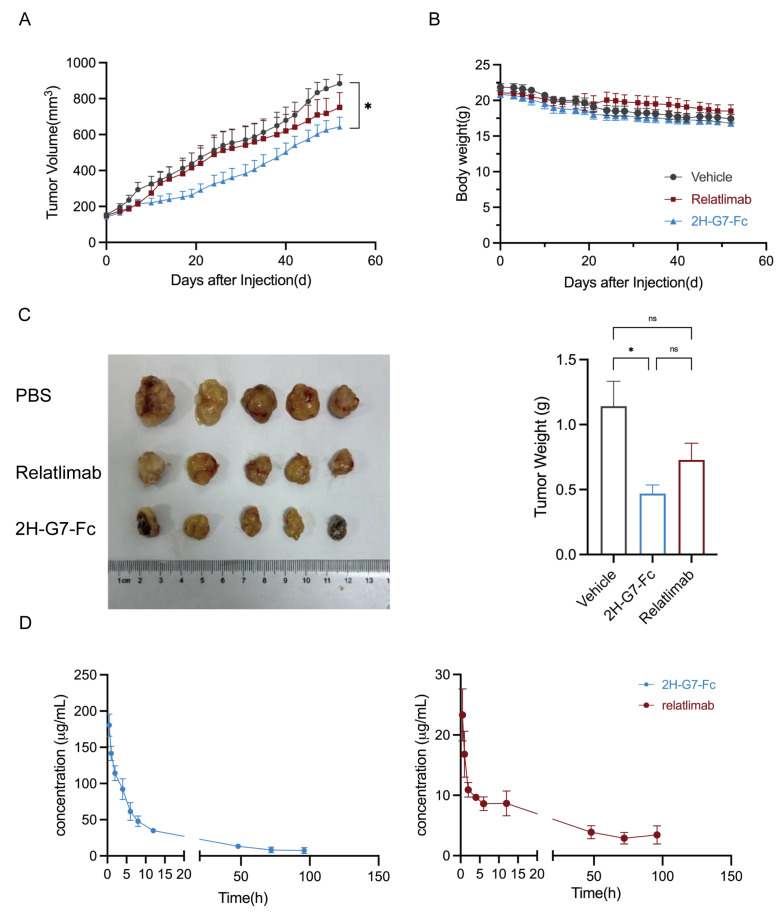
Anti-tumor efficacy of 2H-G7-Fc in a huPBMC-NOG-dKO humanized mouse model of A549 non-small-cell lung cancer. (**A**) Tumor volume and (**B**) body weight growth curves of mice treated with vehicle (PBS), relatlimab (positive control, 10 mg/kg), or 2H-G7-Fc (10 mg/kg) via tail vein injection every four days for 8 weeks; data are presented as the mean tumor volume ± standard error of the mean (SEM). (**C**) Tumor weights of excised tumors at the experimental endpoint; 2H-G7-Fc treatment results in significantly reduced tumor weight compared with the PBS control. * *p* < 0.05, ns, no significance, by one-way ANOVA. (**D**) Pharmacokinetic profiles of 2H-G7-Fc and relatlimab in Balb/C mice. Mice were intravenously administered a single dose of 10 mg/kg 2H-G7-Fc or relatlimab, respectively. Serum antibody concentrations were measured at the indicated time points, and pharmacokinetic parameters were calculated by non-compartmental analysis. Data are presented as mean ± SEM (*n* = 3 per group). Key pharmacokinetic parameters including Cmax, AUC_0−t_, and t_1/2_ are summarized in Table 2.

When subcutaneous tumor volumes reached 100–150 mm^3^, mice were randomly assigned to three groups (*n* = 5 per group) and treated with 2H-G7-Fc, the clinical reference antibody relatlimab (positive control), or PBS (vehicle control) via tail vein injection every four days for 8 weeks. Tumor volumes were measured weekly, and tumor weight was determined at the experimental endpoint. As shown in Figure 6A, administration of 2H-G7-Fc resulted in a delay in tumor growth compared with the PBS-treated vehicle control group throughout the treatment period. Consistent with the tumor volume data, excised tumors from the 2H-G7-Fc group exhibited lower weight compared with the PBS group at the experimental endpoint (Figure 6C), and no significant treatment-related toxicity was observed in any group, as indicated by stable body weight throughout the study (Figure 6B). At the treatment endpoint, tumors were collected and processed into single-cell suspensions for flow cytometric analysis of tumor-infiltrating effector T cells. As shown in Figure S3, substantial infiltration of both CD4+ and CD8+ T cells was observed in tumor tissues. Notably, relatlimab treatment resulted in a relatively higher degree of effector T-cell infiltration than 2H-G7-Fc treatment. These data demonstrate that 2H-G7-Fc possesses potent and specific anti-tumor activity in an immunocompetent humanized NSCLC model, which is mediated by the restoration of T cell-mediated anti-tumor immune responses through effective LAG-3 blockade.

### 3.7. Pharmacokinetic (PK) Analysis of 2H-G7-Fc

To compare the pharmacokinetic (PK) profiles of 2H-G7-Fc and relatlimab in mice, each antibody was administered intravenously at a single dose of 10 mg/kg, and serum antibody concentrations were measured at designated time points. As summarized in Figure 6D and Table 2, 2H-G7-Fc exhibited a significantly higher peak serum concentration (Cmax = 180.56 μg/mL) compared with relatlimab (Cmax = 23.3 μg/mL). Correspondingly, the total systemic exposure (AUC_0−t_) of 2H-G7-Fc reached 2152.03 μg·h/mL, which was markedly greater than that of relatlimab (497.85 μg·h/mL). Although the terminal half-life (t_1/2_) of 2H-G7-Fc was shorter (32.14 h) than that of relatlimab (52.75 h), its substantially higher Cmax and overall AUC likely contributed to its superior in vivo antitumor efficacy observed in the tumor model.

## 4. Discussion

The development of immune checkpoint inhibitors (ICIs) has marked a paradigm shift in the clinical treatment of cancer, providing durable therapeutic responses for a subset of patients with advanced malignancies. However, the clinical benefits of current ICIs remain limited, with a large proportion of patients exhibiting primary or acquired resistance, underscoring the urgent need for novel ICI agents with differentiated mechanisms of action and improved pharmacological properties [1]. In this study, we report the development of a panel of single-domain antibodies (sdAbs) targeting LAG-3—a critical co-inhibitory immune checkpoint receptor—and identify 2H-G7 as a high-affinity lead candidate with potent functional activity and superior in vivo anti-tumor efficacy. Our findings demonstrate that 2H-G7 not only exhibits robust *in vitro* and in vivo activity but also leverages the intrinsic advantages of the sdAb platform, highlighting its potential as a novel agent for next-generation LAG-3-targeted tumor immunotherapy.

The sdAb platform itself confers distinct pharmacological advantages that may translate into improved clinical efficacy compared with conventional monoclonal antibodies (mAbs). Conventional mAbs have a large molecular weight (~150 kDa), which severely limits their penetration into solid tumor tissues and their ability to reach tumor-infiltrating lymphocytes (TILs) within the TME—a major barrier to the efficacy of current ICIs in solid tumors. In contrast, sdAbs such as 2H-G7 have a small molecular weight (~15 kDa), which enables superior tissue and tumor penetration, allowing the antibody to efficiently reach TILs and exert its blocking effect within the TME. Additionally, sdAbs exhibit high thermal and structural stability, excellent solubility, and low immunogenicity in humans, owing to their fully human sequence in our study. They are also amenable to modular genetic engineering, enabling the construction of bispecific antibodies, antibody–cytokine fusion proteins, or other novel formats to further enhance their anti-tumor activity. In this study, we fused 2H-G7 to a human IgG1 Fc fragment to generate 2H-G7-Fc, a pragmatic modification to prolong the serum half-life of the sdAb and enhance its in vivo stability [22]. Notably, the core binding moiety of 2H-G7 retains all the favorable biophysical properties of the sdAb format, providing a versatile scaffold for further engineering and optimization to generate next-generation therapeutic formats.

A key strategic innovation of our study is the successful development of an sdAb that concurrently blocks both the FGL1-LAG-3 and MHC-II-LAG-3 signaling axes. The identification of FGL1 as a major high-affinity ligand of LAG-3 has redefined our understanding of LAG-3-mediated immune suppression [10]. However, most clinical-stage LAG-3-blocking antibodies, including the FDA-approved relatlimab, were initially developed and characterized based on their ability to block the MHC-II-LAG-3 interaction, with FGL1 blockade as a secondary property [21,23]. Notably, the binding sites of MHC-II and FGL1 on LAG-3 are both located within the D1D2 extracellular domains, adjacent but structurally distinct, with partially overlapping functional interfaces rather than fully coincident pockets; MHC-II primarily engages the Loop1 region of D1, while FGL1 binds the neighboring Loop2 region, and these interactions can be genetically separated by site-directed mutagenesis [11]. Relatlimab binds an epitope on D1 near Loop1 that directly overlaps with the MHC-II interface and sterically or allosterically interferes with FGL1 binding, thereby achieving dual blockade of both ligands. In contrast, our sdAbs were selected through a functional screening strategy that prioritizes the inhibition of both LAG-3-ligand interactions, and our results confirm that 2H-G7 potently blocks both the FGL1-LAG-3 and MHC-II-LAG-3 interactions *in vitro*. Importantly, 2H-G7 and relatlimab showed no mutual competition in binding to LAG-3, yet both antibodies potently blocked the interactions of LAG-3 with MHC-II and FGL1. This observation is mechanistically consistent and reliable with the structural topology of LAG-3: 2H-G7 recognizes a unique epitope on D1D2 distinct from that of relatlimab, yet positioned to simultaneously disrupt both ligand-binding interfaces. This dual-blockade profile is of significant clinical importance, as both FGL1 and MHC-II are widely expressed within the complex tumor microenvironment (TME) of most solid malignancies, including NSCLC. Concurrent blockade of both signaling axes by 2H-G7 may therefore achieve a more comprehensive disruption of LAG-3-mediated immune suppression, leading to more robust restoration of anti-tumor T cell function compared with antibodies targeting only a single LAG-3-ligand interaction. Our findings thus identify 2H-G7 as a structurally differentiated LAG-3 inhibitor with a dual-blockade mechanism independent of relatlimab, supporting its potential as a novel candidate for tumor immunotherapy.

Remarkably, in vivo studies demonstrated that 2H-G7-Fc exhibited superior antitumor efficacy compared with the clinically approved anti-LAG-3 antibody relatlimab in a humanized NSCLC mouse model, as evidenced by significantly reduced tumor volume and weight without obvious treatment-related toxicity. The enhanced therapeutic effect of 2H-G7-Fc can be attributed to multiple complementary mechanisms. First, 2H-G7 binds to the D1D2 domains of LAG-3 with high affinity and simultaneously blocks both the FGL1–LAG-3 and MHC-II–LAG-3 signaling axes, thereby more effectively relieving LAG-3-mediated T-cell inhibition and restoring antitumor immune responses. Second, the smaller molecular size of 2H-G7-Fc relative to full-length IgG antibodies such as relatlimab enables more uniform distribution and deeper penetration into solid tumor tissues, promoting more efficient target engagement with LAG-3-expressing tumor-infiltrating lymphocytes in the tumor microenvironment. Third, the high stability and low immunogenicity inherent to the single-domain antibody format support prolonged in vivo activity and an improved safety profile, further enhancing its therapeutic index. Fourth, pharmacokinetic analysis revealed that despite a shorter terminal half-life (32.14 h vs. 52.75 h for relatlimab), 2H-G7-Fc achieved markedly higher peak serum concentration (Cmax) and total systemic exposure (AUC), which likely contributed directly to its enhanced antitumor activity. Collectively, the superior performance of 2H-G7-Fc against a clinically validated benchmark antibody highlights its promising therapeutic potential and validates our functional screening strategy for identifying optimized anti-LAG-3 single-domain antibodies.

Despite the promising findings of this study, several limitations warrant further investigation. First, the in vivo anti-tumor efficacy of 2H-G7 was evaluated in a single humanized NSCLC mouse model; future studies will assess its activity in additional preclinical models of different malignancies, including melanoma and colorectal cancer, to confirm its broad anti-tumor activity. Despite the promising anti-tumor efficacy of 2H-G7-Fc observed in this humanized model, several critical limitations related to species differences in LAG-3 biology and sdAb pharmacokinetic characteristics should be noted for future clinical translation. Interspecies variations in immune checkpoint structure, ligand binding affinity, and tissue distribution can significantly affect the predictive value of preclinical animal models for human outcomes. Although human LAG-3 was targeted in this study, the mouse tumor microenvironment exhibits inherent differences in immune cell composition, cytokine milieu, and LAG-3 expression regulation compared with human patients, which may lead to discrepancies between preclinical efficacy and clinical responses. Second, the molecular mechanisms underlying the superior anti-tumor efficacy of 2H-G7 require further elucidation, including detailed analyses of its effects on T cell subsets, immune cell infiltration into the TME, and the expression of other immune checkpoint molecules. Third, while 2H-G7-Fc exhibits favorable in vivo activity, further engineering of 2H-G7 into novel formats—such as bispecific antibodies targeting LAG-3 and PD-1/PD-L1, or antibody–cytokine fusion proteins integrating IL-15 or IL-2—may further enhance its anti-tumor activity by combining LAG-3 blockade with additional immunostimulatory effects [24]. Fourth, single-domain antibodies (sdAbs) are known to display rapid systemic clearance and short serum half-life in rodents due to their small molecular size and lack of neonatal Fc receptor (FcRn) recycling in some mouse strains, whereas pharmacokinetic profiles in non-human primates and humans are often more favorable [25]. Therefore, the in vivo efficacy and exposure data obtained from the current mouse model may not fully reflect the pharmacokinetic behavior and therapeutic window of 2H-G7-Fc in humans. Finally, systematic preclinical pharmacokinetic and toxicological studies of 2H-G7 in higher species are warranted to support its advancement into clinical development.

In conclusion, this study reports the development and characterization of 2H-G7, a novel high-affinity single-domain antibody targeting the D1D2 domains of LAG-3 with potent dual blockade activity against both FGL1-LAG-3 and MHC-II-LAG-3 interactions. 2H-G7 effectively restores T cell activation *in vitro* and exhibits superior in vivo anti-tumor efficacy compared with the clinical benchmark antibody relatlimab in a humanized NSCLC mouse model, with no detectable treatment-related toxicity. The unique combination of potent dual-ligand blockade activity and the inherent pharmacological advantages of the sdAb platform makes 2H-G7 a promising lead candidate for the development of next-generation LAG-3-targeted tumor immunotherapies. Future work will focus on optimizing the therapeutic format of 2H-G7 and advancing it through preclinical development for potential clinical translation.

## Figures and Tables

**Table 1:** Equilibrium dissociation constants (K_D_) of the antibodies by BLI.

	K_on_ (1/Ms)	K_off_ (1/s)	K_D_ (M)
2H-C10	8.34 × 10^3^	6.32 × 10^-4^	7.58 × 10^-8^
2H-G7	3.15 × 10^4^	6.26 × 10^-4^	1.99 × 10^-8^
H-C4	9.05 × 10^4^	3.08 × 10^-3^	3.41 × 10^-8^

**Table 2:** Pharmacokinetic parameters of antibodies in tumor-xenograft mice.

	Cmax (μg/mL)	AUC_0−t_ (μg⋅h/mL)	t_1/2_ (h)
2H-G7-Fc	180.560	2152.03	32.14
Relatlimab	23.3	497.85	52.75

## Data Availability

The original contributions presented in this study are included in the article/Supplementary Materials. Further inquiries can be directed to the corresponding author.

## Supplementary Materials

The following supporting information can be downloaded at: https://www.mdpi.com/article/10.3390/cimb48050478/s1.
