# Supplementary material for: A Potent Single-Domain Antibody Targeting LAG-3 for Efficient Tumor Immunotherapy
Source: Curr Issues Mol Biol. 2026 May 4;48(5):478. doi: 10.3390/cimb48050478 (PMC13204672; doi:10.3390/cimb48050478)
Supplement: Supplementary file 1 [file cimb-48-00478-s001.zip › cimb-4193138-supplementary.pdf]

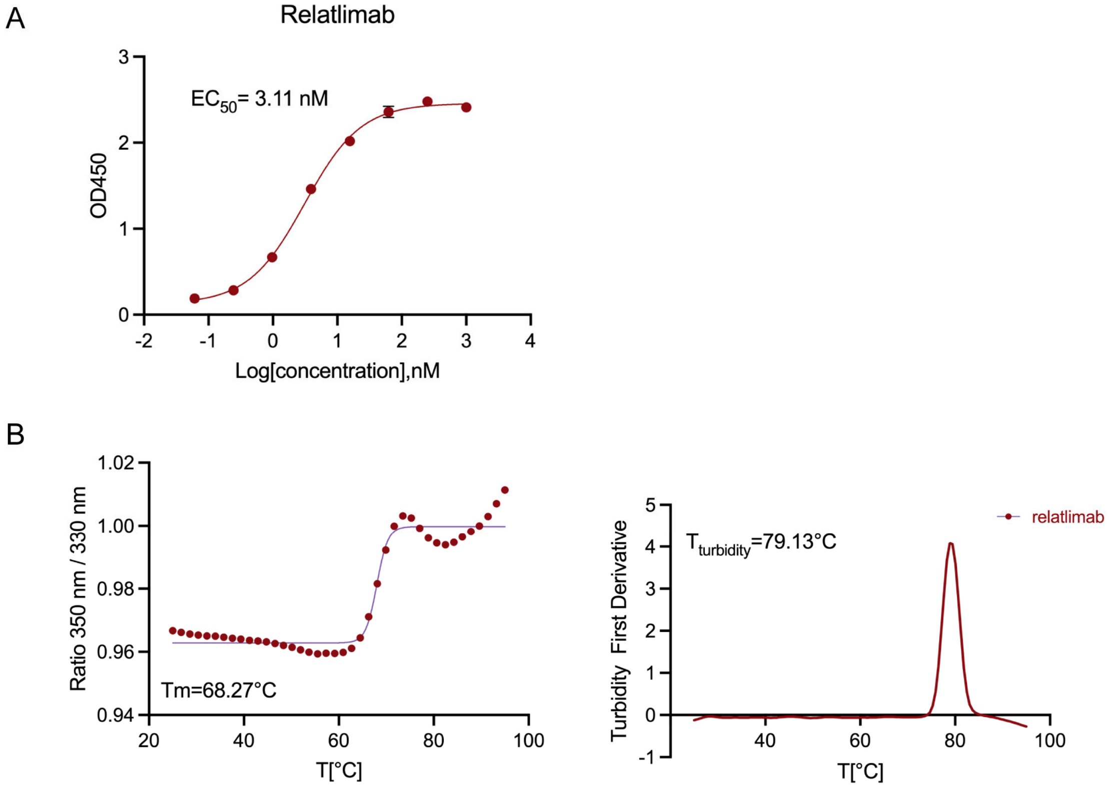

Figure S1. Biological properties of Relatlimab. (A) ELISA analysis of Relatlimab, n = 3 independent experiments. (B) Thermal stability of Relatlimab as measured by Prometheus Panta (NANOTEMPER, Germany). Thermal stability of the antibody was assessed by nano differential scanning fluorimetry (nanoDSF) based on intrinsic tryptophan fluorescence. Samples were heated from 20 °C to 100 °C, and the fluorescence intensity ratio (F350/F330) was recorded as a function of tem-perature. Data represent the mean ± SD of three independent experiments. Antibody aggregation was concurrently monitored by backreflection, and the aggregation tempera-ture (Tagg) was determined from the first derivative of the backreflection signal. Data rep-resent the mean ± SD of three independent experiments.

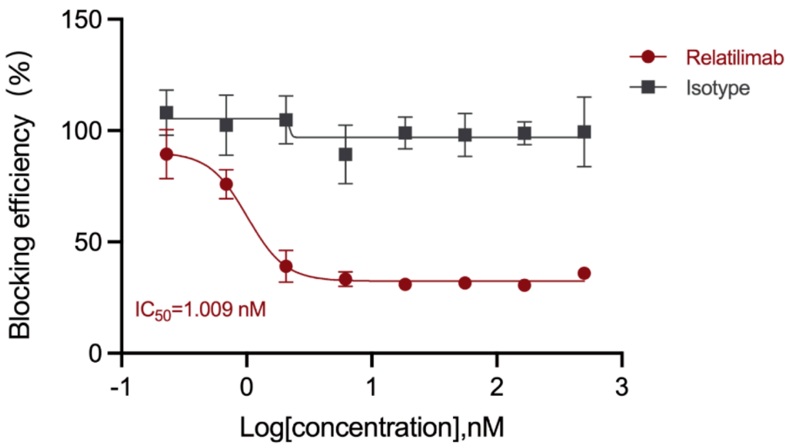

Figure S2. Competitive flow cytometry assay to evaluate the inhibition of LAG3-MHC-II binding on Raji cells by the Relatlimab.

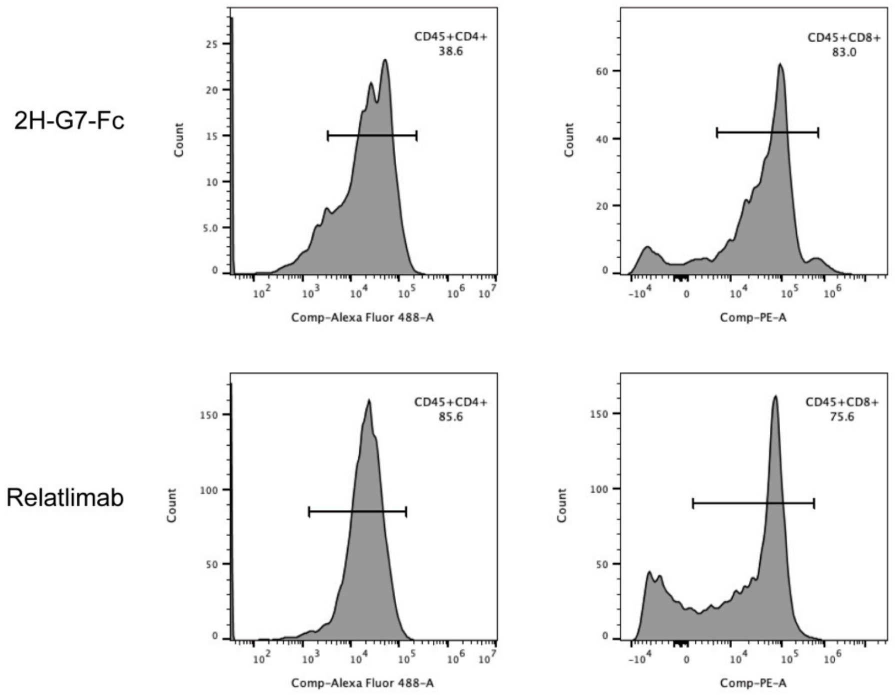

Figure S3. Analysis of tumor-infiltrating effector T cells.

Tumors were dissociated into single-cell suspensions, followed by staining with anti-CD45, anti-CD4, and anti-CD8 antibodies for 30 min on ice. After washing three times, flow cytometric analysis was performed to evaluate tumor-infiltrating CD4+ and CD8+ T cells. n = 3 independent experiments.
